# Influence of Chronotype on Cycling Performance in Simulated 20‐km Time Trials—A Pilot Study

**DOI:** 10.1111/jsr.70268

**Published:** 2025-12-16

**Authors:** Sabrina Forster, Sascha Schwindling, Chris Abbiss, Fabienne Döringer, Andreas Klütsch, Anne Hecksteden, Tim Meyer

**Affiliations:** ^1^ Institute for Sport and Preventive Medicine Saarland University Saarbrücken Germany; ^2^ Olympic Training Center Rheinland‐Pfalz/Saarland Saarbrücken Germany; ^3^ School of Medical and Health Sciences Edith Cowan University Joondalup Australia; ^4^ Institute of Sports Science, Johannes‐Gutenberg University Mainz Germany; ^5^ German Triathlon Union Frankfurt Germany; ^6^ Institute of Sport Science University of Innsbruck Innsbruck Austria; ^7^ Institute of Physiology, Medical University of Innsbruck Innsbruck Austria

**Keywords:** circadian rhythm, pacing, performance, sleep

## Abstract

The chronotype (CT) refers to an individual's diurnal preference towards morningness (M) or eveningness (E). The aim of this study was to determine the influence of chronotype on 20‐km cycling performance throughout the day. Seventy‐six competitive male cyclists and triathletes completed the Morningness‐Eveningness Questionnaire to determine chronotype. Only participants categorised as ‘definite’ M‐ (*n* = 10) and E‐types (*n* = 7) were included in the study. In a randomised order and separated by 2–7 days, participants performed four self‐paced 20‐km cycling time trials at four different times of the day (06:00 h, 12:00 h, 18:00 h, 22:00 h). Mental readiness was assessed before each trial. Performance across all participants was significantly better in the evening compared to the morning (change: 2.1% ± 3.8%; *p* = 0.008). Related to individual's mean performance E‐types performed significantly better in the evening compared to the morning (*p* = 0.02). Specifically, athletes were 40 s faster at 18:00 h compared to 06:00 h. Mental readiness in E‐type athletes was significantly lower at 06:00 h compared to all other times (*p* < 0.04). The present study indicates that E‐type athletes perform better later in the day. This might be important for the scheduling of training times and the preparation for competition, especially in the morning.

## Graphical abstract

Evening‐type athletes show improved cycling performance later in the day, whilst morning types peak earlier. These differences appear linked to variations in mental readiness and sleepiness, highlighting the importance of considering chronotype in performance planning.

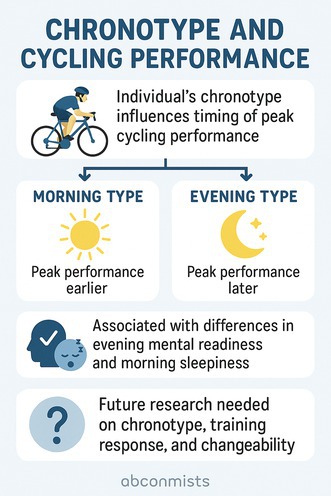

## 1 Introduction

Diurnal variation in athletic performance has been of interest for several decades, with research indicating that physical performance is generally better in the evening compared with the morning (Bommasamudram et al. 2022; Pullinger et al. 2020; Ravindrakumar et al. 2022). At the individual level, peak performance might further be influenced by circadian phenotypes and chronotype (Anderson et al. 2018; Brown et al. 2008; Ingram et al. 2016). Chronotype refers to an individual's predisposition towards morningness or eveningness (Vitale and Weydahl 2017) and is usually assessed subjectively, based on the time‐of‐day at which individuals feel alert, sleepy, vigorous, or fatigued and when they prefer to be asleep, awake, or perform mental or physical tasks (Brown et al. 2008; Rae et al. 2015; Roenneberg et al. 2007). Based on these self‐assessments, individuals are classified as ‘morning types’, ‘intermediate’ or ‘neither types’ and ‘evening types’ (Horne and Ostberg 1976). Morning type individuals are characterised by early wake times and peak alertness during mid‐morning, whereas evening‐types show delayed wake times and peak alertness during the late afternoon or evening (Anderson et al. 2018).

It has been found that chronotype may be influenced by genetics, with several studies identifying particular genetic variants associated with diurnal preference (Anderson et al. 2018; Archer et al. 2003; Chang et al. 2019; Ebisawa et al. 2001; Kunorozva et al. 2012). Additionally, several studies have observed differences between chronotypes in the diurnal rhythm of several physiological systems associated with performance, with differences in markers such as plasma levels of hormones (e. g. cortisol, melatonin), glucose tolerance, body temperature, and blood pressure (Baehr et al. 2000; Bailey and Heitkemper 2001; Rae et al. 2015). For example, melatonin peaks around three hours later in evening types compared with morning‐type individuals, often leading to later bed‐ and awakening‐times (Adan et al. 2012). Understanding the influence of the individual diurnal rhythm has been of interest for coaches and athletes, as intra‐individual differences in internal physiology are likely to impact athletic performance (Anderson et al. 2018; Silveira et al. 2020). Previous research observed that endurance athletes with extreme morning or evening phenotypes tend to perform better near their circadian peak (Anderson et al. 2018; Brown et al. 2008; Facer‐Childs and Brandstaetter 2015; Rae et al. 2015). Considering that these individual characteristics of exercise performance possess their specific diurnal rhythms (Hill et al. 1992; Souissi et al. 2007), time of day and/or chronotype might also influence pacing selection. Pacing is defined as the self‐regulation of speed throughout an exercise and is considered extremely important in athletic performance (Abbiss and Laursen 2008). However, to the authors' knowledge, there is only limited research available evaluating a potential influence of time of day and/or chronotype on pacing selection during prolonged high‐intensity exercise.

Whilst an athlete's preferred time‐of‐day for exercise may be driven by the athlete's individual diurnal rhythm, factors such as work, study, family and access to training facilities also influence their actual and habitual training time. Furthermore, competitions are obviously scheduled at different times during the day without considering diurnal variations in performance. For example, major competitions (e.g., World championships, Olympic Games finals) are often scheduled in the evening due to television requirements. Available research indicates that the habitual training time may shift peak performance time towards the time‐of‐day training takes place (Brown et al. 2008; Chtourou and Souissi 2012). As such, it is speculated that habitual training time might diminish potential negative effects on performance from one's internal circadian rhythm (Chtourou and Souissi 2012).

The current research evaluating the potential effects of individual circadian rhythms is inconclusive and there is a lack of research in endurance‐based sports evaluating chronotype effects on sport‐specific performance. Specifically, evaluating time trial performance could provide a deeper understanding as these tests are close to real competitions whilst being highly standardised. Therefore, the aim of the current study was to evaluate differences in 20‐km cycling time trial performance between morning and evening chronotypes. Based on current findings it was hypothesised that performance of evening types will be better in the evening compared with the morning, and that performance of morning types will be better in the morning.

## 2 Materials and Methods

To consider practical relevance the effect size for the sample size calculation was based on the mean difference (md) between the 1st and 4th place during the German Road Cycling Championship between 2019 and 2021 (md: 66.5 s) and the variability of laboratory based cycling (Laursen et al. 2003). A sample size calculation revealed that 38 cyclists will be necessary to observe a significant interaction between chronotype and performance with a power of 80% (G*power; *f* = 0.375, alpha = 0.05, power = 0.80, corr = 0.5). A total of 70 ‘trained/developmental’ male cyclists and triathletes as classified by the ‘Participation Classification Framework’ (McKay et al. 2022) were recruited for the study and completed the Morningness‐Eveningness Questionnaire (Horne and Ostberg 1976) to differentiate between chronotypes. Only those athletes categorised as either definite morning‐ (M‐types; *n* = 10) or definite evening‐types (E‐types; *n* = 7) completed the performance test (described below) and were included in the final analysis resulting in *n* = 17 overall (mean ± SD) and [range]: age 37.9 ± 10.3 [22–55] years, body mass = 77.9 ± 7.6 [64–91] kg, height = 181.0 ± 4.9 [174–192] cm, training volume = 9550 ± 2924 [4.000–15.000] km/y, maximum Power output from incremental test = 356 ± 45 [275–423] W (4.6 ± 0.5 [3.8–5.8] W/kg) and habitual total sleep time = 8.7 ± 0.7 [7–9.5] h. Inclusion criteria was > 2 years of cycling competition experience, injury‐free with no diagnosed sleep disorders and not completed shiftwork or travel outside the local time‐zone in the past month. Participants had to be healthy males (18–30 years), habitually retire between 22:00–23:30 h and rise at 06:00–07:30 h and agree to retire to bed at 22:30 and rise at 06:30 h; which is not too dissimilar to their natural sleep patterns. None of the participants were receiving any pharmacological treatment (including non‐steroidal anti‐inflammatory drugs, NSAIDs) throughout the study period. Habitual caffeine consumption was assessed using the caffeine consumption questionnaire (CCQ) and those with > 150 mg per day were excluded (Drust et al. 2005). Further, all participants self‐reported no preference to training regarding time of day.

The study was conducted in accordance with the Declaration of Helsinki and was approved by the local Human Research Ethics Committee (Ärztekammer des Saarlandes, Saarbrücken, Germany). The design followed the checklist of considerations in chronobiological studies on human and sporting performance by Edwards et al. (2024). Before testing, all participants gave written informed consent and provided details on their training and racing history. All cyclists had to undertake a health examination (including history, physical examination, and resting ECG) prior to participating in the study. Participants attended the laboratory on five separate occasions. The room was held at a dry temperature, humidity, barometric pressure and ambient light of 19°C, 35%–45%, 750–760 mmHg and 750 lx, respectively for all sessions. Only one person completed the time trial in the room at a given time.

During the first visit participants performed an incremental cycling test to exhaustion on an electromagnetic cycle ergometer to determine peak power output (Cyclus 2 ergometer, RBM electronic‐automation GmbH, Leipzig). Power output was progressively increased by 50 W every 3 min until volitional exhaustion. On separate days following this, and in a randomised counterbalanced order to minimise any potential learning effects (Monk and Leng 1982), participants performed four self‐paced 20 km cycling time trials at different times of the day: 06:00 h, 12:00 h, 18:00 h, 22:00 h. It was ensured that the order of tests was equally distributed between the participants. Familiarisation trials were not included as previous research observed no difference in performance between three 20‐km time trials (Thomas et al. 2012). Coefficient of variations were below 5% between the three trials (trial 1 vs. trial 2: 1.6%, trial 2 vs. trial 3: 2.2%) (Thomas et al. 2012), indicating little measurement error in time trial performance in trained participants (Atkinson and Nevill 1998). Considering the high number of laboratory visits (5 overall), familiarisation trials were not included. Participants were asked to refrain from strenuous exercise in the 48 h prior to each trial and then replicate their training load and nutrition (including caffeine) from the first trial as closely as possible before each other trial (controlled by means of written training diaries). Water and non‐caffeinated/non‐alcoholic, calorie‐free beverages were allowed ad libitum. The participants were free to live a ‘normal life’, between sessions sleeping at home at night; and attending lectures and doing light office work in the day. All time trials were performed in standardised laboratory conditions and were completed on a valid (Reiser et al. 2000) electromagnetically braked cycle ergometer (Cyclus 2 ergometer, RBM electronic‐automation GmbH, Leipzig). The ergometer was equipped with a racing bike frame and the axles allowing movement to replicate a natural cycling movement. Participants adjusted the ergometer to their preferred racing position (which was recorded and replicated for each trial) and wore their own cycling shoes and used their own pedals.

Participants performed the Lamberts and Lambert submaximal cycling test (LSCT) as a standardised warm‐up (5 min at 60% of HR_max_, 6 min at 80% of HR_max_ and 3 min at 90% of HR_max_; (Lamberts et al. 2011)), after which they were given 5 min to relax and prepare for the subsequent trial. A flat 20 km time trial profile was created using the Cyclus2 software and was used for all trials. Power output was averaged over every 2 km. Participants were instructed to complete the 20 km distance as fast as possible. Visual feedback on the distance covered, power, pedalling frequency and heart rate was available during all trials. The same range of electronic gear ratios was used for each trial and the participant started each trial with the same gear ratio; they were permitted to adjust this throughout the trial to realise their preferred cadence. Water consumption was ad libitum during each time trial. All trials were supervised by the same experimenter and standardised verbal encouragement was provided during the last split in each trial. A recent meta‐analysis recommended using standardised verbal encouragement in laboratory‐based studies to ensure that participants give maximum effort during an endurance task (McCormick et al. 2015). The experimenter provided consistent, brief motivational cues (e.g., ‘keep it up,’ ‘push through,’ ‘stay strong’) at predetermined intervals, ensuring that all participants received the same type and intensity of encouragement during the end‐spurt. An electrical fan was positioned approximately 0.5 m in front of the ergometer during each trial.

Gas exchange parameters (VO_2_, VCO_2_, respiratory exchange ratio (RER)) were measured continuously using an automated online metabolic cart (Cortex, Meta Lyzer 3, Leipzig, Germany). The gas analyser and flow turbine were calibrated before each test using a certified standard gas (15.0% O_2_, 5% CO_2_) and ambient air as well as a 3‐L syringe for volume calibration (Hans Rudolph, Kansas City, USA). Capillary whole‐blood samples (20 μL) were taken from the hyperemized earlobe immediately, 2, 3, 5 and 7 min after exercise and analysed for peak blood lactate concentration (BLa; automated enzymatic‐amperometric method, Greiner BioChemica, Flacht, Germany). Heart rate (HR) was continuously recorded and analysed using PolarPro Trainer 5 (Polar Electro, Kempele, Finnland).

Prior to each trial, participants rated their mental readiness, sleepiness, and motivation on 0–100 visual analogue scales. During each trial, participants were asked to report their perceived exertion (RPE) using Borg's 0–10 scale (Borg 1982; Foster et al. 2001) at 2 km intervals. Prior to the experimental trials, participants were given standard instructions for overall RPE and were asked to report based on the degree of whole body heaviness and strain experienced during the exercise task (Borg 1982). Participants were also familiarised with the RPE scale during the incremental test, during which the low and high anchor points were established using standard procedures (Borg 1982). Since it has recently been shown that the presence of a male or female observer has a significant influence on RPE (Winchester et al. 2012), participants were asked to report their RPE to the same investigator in each test.

To allow for individual interpretation in performance changes, a Z‐score was calculated using the mean power and standard deviation of all four trials per participant. Physiological (HR, VO_2_, RER), performance data, and RPE are reported as means and standard deviations (SD) as they were normally distributed (Shapiro–Wilk test). As perceptual data (VAS) and Z‐score were not normally distributed, median and interquartile ranges are used for display. A mixed repeated measures ANOVA (*lm* package in RStudio) was performed to examine differences in performance as well as mean physiological (HR, VO_2_, RER) and perceptual responses (RPE) (within‐subject factor: Time (6 am, 12 pm, 6 pm, 10 pm); between‐subject factor: Chronotype (M/E)). To compare pacing, velocity in all trials was expressed relative to average race velocity (normalised mean velocity). This approach of expressing pacing as the difference between current and overall mean velocity is widely accepted (Abbiss and Laursen 2008). A mixed repeated measures ANOVA was performed to examine differences in pacing (within‐subject factor 1: Time (6 am, 12 pm, 6 pm, 10 pm); within‐subject factor 2: split (km 1 to 10); between‐subject factor: Chronotype (M/E)). When significant main effects were observed, a Tukey post hoc test (*lm* package) was performed. *p* < 0.05 for the α error was accepted as the level of significance for statistical comparison. Partial *η* ^2^ was used to calculate effect sizes for the ANOVA results. Additionally, Cohen's effect sizes (*d*; including 95% confidence intervals) and thresholds (0.2, 0.5 and 0.8 for small, moderate and large (Cohen 1988)) were also used to compare the magnitude of the changes in overall performance parameters. All statistical analyses were conducted using RStudio (Version 1.4.1717, RStudio Inc.).

## 3 Results

### 3.1 Time of Day

No order effect was observed for either performance (*p* > 0.92) or subjective (*p* > 0.13) variables. A significant main effect for time of day was observed for finishing time (*F*(3, 59) = 4.3, *p* = 0.008) with better performances at 06:00 h compared to 22:00 h. No significant main effect for time of day was observed in any other measured parameter (*P*_mean_: *F*(3, 59) = 0.09, *p* = 0.97; HR_mean_: *F*(3, 60) = 0.36, *p* = 0.78; RPE_mean_: *F*(3, 60) = 0.54, *p* = 0.66; VO_2mean_: *F*(3, 54) = 0.35, *p* = 0.79; RER_mean_: *F*(3, 54) = 0.74, *p* = 0.53, Table 1).

**TABLE 1:** Overall results (*n* = 17) for time of day (physiological markers and RPE are displayed as means ± standard deviation, perceptual responses from VAS scales as median with interquartile ranges (25th and 75th)).

	06:00 h	12:00 h	18:00 h	22:00 h
*P*_mean_ (W)	250.5 ± 48.8	252.8 ± 49.6	250.5 ± 46.2	258.1 ± 46.1
Finishing time (min: sec)	32:12 ± 2:22	32:07 ± 2:22	31:59 ± 2:08	31.35 ± 2:33
HR_mean_ (bpm)	170.9 ± 7.4	173.4 ± 8.6	171.9 ± 8.1	173.2 ± 7.8
RPE_mean_ (AU)	6.4 ± 0.9	6.5 ± 1.1	6.5 ± 0.9	6.7 ± 1.0
VO_2mean_ (mL/min/kg)	46.4 ± 8.0	44.4 ± 6.3	45.4 ± 7.1	46.9 ± 7.4
RER_mean_	1.04 ± 0.03	1.06 ± 0.04	1.06 ± 0.05	1.06 ± 0.05
Sleepiness (AU)	3.2 (1.8/6.4)	2.9 (1.5/4.8)	3.4 (1.7/7.5)	4.9 (2.3/6.5)
Motivation (AU)	6.2 (5.1/7.3)	6.5 (5.5/8.0)	6.4 (3.0/8.4)	6.6 (4.8/8.0)
Mental readiness (AU)	4.9 (3.8/7.6)	7.0 (6.0/8.2)	6.7 (4.2/8.4)	5.9 (3.3/7.7)

### 3.2 Chronotype

**FIGURE 1:**
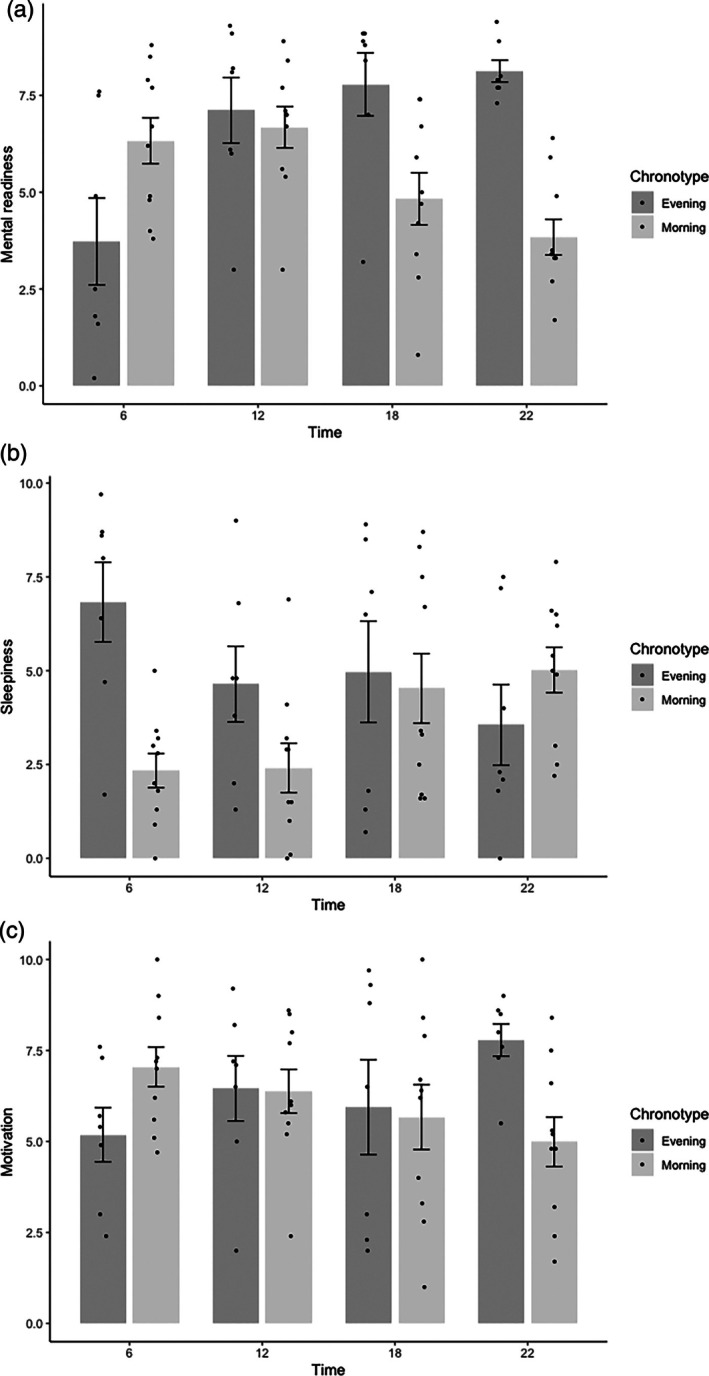
Median with interquartile ranges of Mental readiness (upper left graph), Sleepiness (upper right graph), and Motivation (lower left graph) for each chronotype (Morning type: Left side, Evening type: Right side) and time of day.

No significant main effect for chronotype was observed for *P*_mean_ (*F*(1, 59) = 0.13, *p* = 0.72), finishing time (*F*(3, 60) = 0.12, *p* = 0.95, *η* ^2^ < 0.001), HR_mean_ (*F*(1, 60) = 1.07, *p* = 0.31, *η* ^2^ = 0.02) and VO_2mean_ (*F*(1, 54) = 0.007, *p* = 0.94, *η* ^2^ < 0.001; Table 2). However, a significant main effect was found for RER_mean_ (*F*(1, 54) = 6.01, *p* = 0.02, *η* ^2^ = 0.10) and RPE_mean_ (*F*(1, 60) = 21.8, *p* < 0.001, *η* ^2^ = 0.26), with post hoc analysis revealing a significantly higher RPE_mean_ in the E‐type group (*p* < 0.05; Table 2). Furthermore, significant interaction effects were observed for mental readiness (*F*(3, 60) = 9.86, *p* < 0.01, *η* ^2^ = 0.33), sleepiness (*F*(3, 60) = 4.2, *p* = 0.01, *η* ^2^ = 0.17) and motivation (*F*(3, 60) = 2.92, *p* = 0.04, *η* ^2^ = 0.13; Figure 1). Post hoc analysis revealed a significantly lower mental readiness at 06:00 h compared to 18:00 h (sleepiness: *p* = 0.006) and 22:00 h (*p* = 0.002) in E‐type athletes. In comparison to M‐types, E‐types felt mentally more ready to perform at 22:00 h (*p* = 0.001). Furthermore, sleepiness was significantly greater at 06:00 h in E‐types compared to M‐types (*p* = 0.01). Post hoc analyses did not reveal effects of time on motivation (*p* > 0.21).

**TABLE 2:** Overall results (*n* = 17) for both morning and evening types (means ± standard deviation).

	Morning types (*n* = 10)				Evening types (*n* = 7)			
	6 am	12 pm	6 pm	10 pm	6 am	12 pm	6 pm	10 pm
*P*_mean_ (W)	253.6 ± 42.3	255.7 ± 40.4	250.8 ± 38.8	258.8 ± 37.2	246.0 ± 60.1	248.7 ± 63.9	249.8 ± 60.8	257.2 ± 59.9
Time (min: sec)	31:59 ± 2:03	31:53 ± 1:56	32:04 ± 1:51	31:19 ± 2:31	32:30 ± 2:54	32:26 ± 3:01	31:15 ± 2:38	31:58 ± 2:46
Z‐score (AU)	0.16 ± 1.04	−0.13 ± 0.74	0.55 ± 0.76	−0.59 ± 0.60	0.75 ± 0.70	0.37 ± 0.58	−0.69 ± 0.66	−0.42 ± 0.81
HR_mean_ (bpm)	170.6 ± 8.6	172.7 ± 9.7	170.2 ± 9.8	172.5 ± 9.5	171.3 ± 5.9	174.5 ± 7.4	174.2 ± 4.6	174.2 ± 4.8
RPE (AU)	6.1 ± 0.9	6.0 ± 1.1	6.1 ± 0.8	6.5 ± 1.1	6.9 ± 0.8**	7.2 ± 0.7**	7.1 ± 0.5**	7.3 ± 0.5**
VO_2mean_ (ml/min/kg)	45.7 ± 6.6	45.7 ± 6.1	45.6 ± 6.5	45.7 ± 5.5	45.7 ± 6.3	47.5 ± 10.8	42.5 ± 6.6	45.2 ± 8.6
RER_mean_	1.03 ± 0.03	1.06 ± 0.03	1.05 ± 0.05	1.05 ± 0.03	1.06 ± 0.04	1.07 ± 0.05	1.08 ± 0.03	1.08 ± 0.06
Mental performance (AU)	6.5 (3.8/7.9)	7.0 (5.9/7.6)	4.9 (3.6/6.5)	3.4 (3.3/4.6)**	2.5 (1.7/6.2)*	8.1 (6.1/8.7)	8.8 (7.7/9.0)	7.9 (7.7./8.5)**
Sleepiness (AU)	2.4 (1.4/3.1)**	2.2 (1.1/3.1)	3.4 (1.9/7.3)	5.2 (3.5/6.4)	8.0 (5.6/8.7)**	4.8 (2.9/5.8)	6.5 (5.6/8.7)	2.6 (2.0/5.6)
Motivation (AU)	7.1 (5.6/8.1)	6.1 (5.6/7.9)	6.3 (3.5/7.6)	5.0 (3.6/6.3)	5.4 (3.7/6.5)	7.1 (5.8/7.7)	6.5 (3.4/8.5)	8.0 (7.5/8.6)

*Note:* Median with 25th and 75th quartile are displayed for mental performance, sleepiness and motivation.

* *p* < 0.05 within E‐types.

** *p* < 0.05 between types.

**FIGURE 2:**
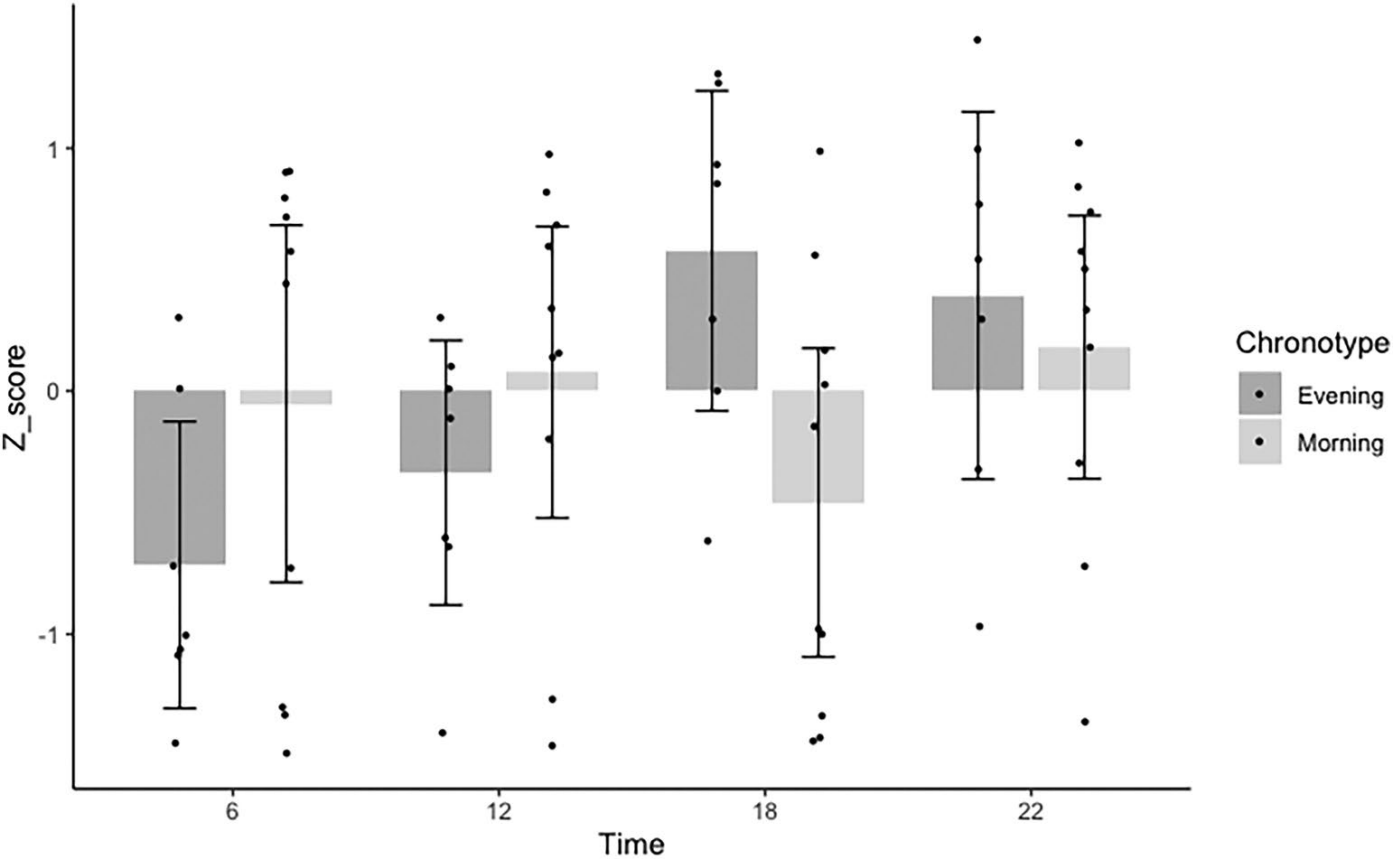
Median with interquartile ranges of the Z‐score for each chronotype (Morning‐type: Left graph, Evening‐type: Right graph) and time of day.

A significant interaction effect was observed for the Z‐score of the individual performance (*F*(1, 60) = 5.13, *p* = 0.02, *η* ^2^ = 0.14). Post hoc analysis revealed that, relative to their average performance, E‐types performed better in the evening than in the morning (06:00 h vs. 18:00 h: *p* = 0.02; Figure 2). As such, overall time in E‐types was on average 39.9 s (±30.9 s) faster at 18:00 h compared to 06:00 h (Table 3). Indeed, all E‐type athletes recorded their best performance during the second half of the day (31:54 vs. 32:28 min), whereas 6 out of 11 M‐types showed their best performance in the first half of the day (31:56 vs. 31:41 min).

**TABLE 3:** Mean difference and effect sizes for total finishing time for both morning (*n* = 10) and evening (*n* = 7) types (means ± standard deviation).

	Morning types				Evening types			
	06:00 h–18:00 h	06:00 h–22:00 h	12:00 h–18:00	12:00 h–22:00 h	06:00 h–18:00 h	06:00 h–22:00 h	12:00 h–18:00	12:00 h–22:00 h
Mean difference (s)	40.5 ± 75.9	−5.4 ± 44.2	−6.2 ± 41.9	34.3 ± 77.8	39.9 ± 30.9	32.5 ± 44.9	−4.2 ± 42.4	28.3 ± 26.6
Cohens *d*	0.04	−0.29	0.10	0.26	0.23	0.19	0.21	0.16

*Note:* A negative value indicates a faster time at the later time of day.

### 3.3 Pacing

**FIGURE 3:**
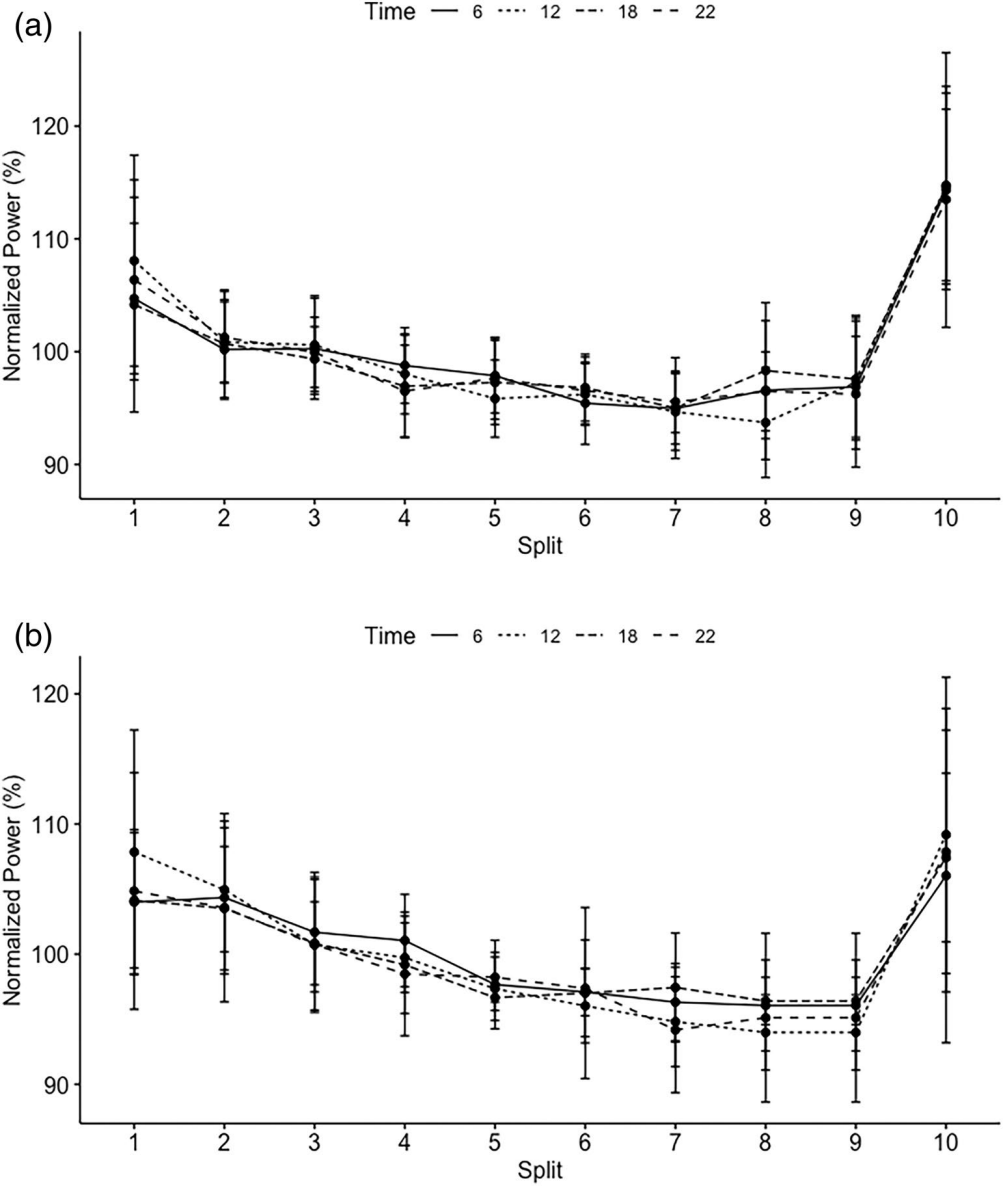
Normalised pacing pattern at 6 am (solid lines), 12 pm (dotted lines), 6 pm (dashed lines) and 10 pm (dashed lines) for Morning types (left graph) and Evening types (right graph).

No significant main effect in the pacing pattern was observed for chronotype (*F*(1, 661) = 0.10, *p* = 0.74) and time of day (*F*(1, 661) = 0.09, *p* = 0.77; Figure 3). However, a significant main effect was observed for split (*F*(1, 661) = 9.71, *p* = 0.002; Figure 3). Within‐group analysis revealed that E‐types showed a significantly faster first (*p* < 0.001) and last split (*p* < 0.001) in all trials, resulting in a parabolic‐shaped pacing pattern. Additionally, split 2 was significantly faster compared to split 5–9 (*p* < 0.001) and split 3 was significantly slower compared to split 8 and 9 (*p* < 0.009). Analysis revealed no within‐group differences between splits in the M‐type group (*p* > 0.05; Figure 3).

## 4 Discussion

The purpose of the present study was to determine the effects of chronotype on 20‐km cycling time trial performance. The findings from the study were that: (i) evening type athletes performed 2% better in the evening compared with the morning, (ii) on average the cyclists in the study performed better in the evening compared to the morning, (iii) no difference in performance was observed for morning type athletes between evening and morning performances, (iv) perceived evening mental readiness and morning sleepiness were greater in evening type compared with morning type athletes.

### 4.1 Chronotype‐Dependent Diurnal Variation in Cycling Performance and Pacing

In the present study we observed a significant difference in cycling performance throughout the day with better average performances across all athletes in the evening compared to the morning. Such findings were expected based upon the current literature indicating that exercise performance tends to be better in the evening compared with the morning (Bommasamudram et al. 2022; Pullinger et al. 2020; Ravindrakumar et al. 2022). When examining individual chronotypes we observed that evening type athletes performed 2.0% better in the evening when compared with the morning; however, no such difference in performance was observed in morning type athletes. Additionally, almost all evening type cyclists (6 out of 7) recorded their best performance during the second half of the day. This observation is in line with research published in other endurance‐based sports. For example, elite swimmers who self‐report as evening types swim faster in the evening, whereas morning types perform better in the morning (Anderson et al. 2018; Rae et al. 2015). Rae et al. (2015) observed faster 200‐m times of evening type swimmers in evening sessions compared to morning sessions, whereas Brown et al. (2008) observed a significant reduction in rowing speed throughout the day in morning type athletes. Furthermore, Facer‐Childs and Brandstaetter (2015) examined the effects of chronotype on a multi‐stage fitness test and observed a diurnal variation in evening type athletes of 26.2% (±3.97%) and only 7.62% (±1.18%) in morning types. In addition, the authors observed that the average peak performance for evening types was 11 h after entrained wake‐up (i.e., the average wake‐up time for the previous 2 weeks), whereas performance in morning types peaked 6.5 h after wake‐up (Facer‐Childs and Brandstaetter 2015). As such, the authors speculate that evening types appear to require greater time for increases in alertness and wakefulness and do not reach maximum performance levels as quickly as morning types after wake‐up (Facer‐Childs and Brandstaetter 2015). It should be acknowledged that participants may have had to wake earlier for the 06:00 h trial, potentially reducing sleep duration and affecting performance (Fullagar et al. 2015). However, this scenario likely reflects the reality of many athletes. For instance, swimmers do not fall asleep earlier when required to rise early for morning training (Sargent et al. 2014), resulting in reduced sleep. Thus, competing under partial sleep restriction may mirror the real‐world demands faced by athletes, particularly those with a late chronotype. Additionally, research evaluating the effect of acute partial sleep loss on endurance performance is inconclusive (Fullagar et al. 2023; Fullagar et al. 2015). From the limited evidence available, it appears that sports involving high cognitive demands, such as quick reaction time and fast decision‐making, are more susceptible to performance reduction following sleep deprivation than sports that rely more on endurance performance (Fullagar et al. 2023).

Our observed 2% variation in performance is likely to be meaningful to competition and training. Indeed, the day‐to‐day variability of laboratory‐based 20‐km cycling performance in recreational cyclists is approximately 2% (Borg et al. 2018; Thomas et al. 2012), whereas within‐season variability of elite road cycling performance ranges between 0.4% and 1.7% (Malcata and Hopkins 2014). Neither time of day nor chronotype seems to have an influence on pacing selection. This is in line with Zadow et al. (2020) who recently demonstrated that time of day had no influence on pacing selection in trained cyclists. As previously discussed, athletes develop a stable pacing pattern throughout their career (Micklewright et al. 2010; Skorski et al. 2014), which seems to be robust against diurnal changes in a variety of physiological systems. However, as only limited data is currently available, more research is needed evaluating the effects of diurnal changes on pacing pattern.

### 4.2 Psychophysiological Readiness Varies by Chronotype

Sleepiness was rated higher at 06:00 h in evening type compared with morning type athletes, supporting the assumption that evening types struggle more with performing outside their diurnal preference (Vitale and Weydahl 2017). Additionally, the better evening performance observed in evening type athletes was accompanied by a significantly higher perceived mental readiness in the evening compared to the morning. A lower perceived mental readiness is likely to influence motivation and potentially performance as has been hypothesised (Vitale and Weydahl 2017). One reason for that might be that the diurnal rhythm of hormonal markers such as melatonin and cortisol seems to be delayed in evening types, which means that they prefer to wake up and go to bed later than other chronotypes (Adan et al. 2012). Greater sleepiness and motivation consequently seem to affect performance early in the morning. Furthermore, evening types generally seem to show greater mood disturbances throughout the day than morning types (Rae et al. 2015). These diurnal effects may be strong enough to influence the distribution of chronotypes in endurance sports that regularly compete in the morning, such as running, cycling, and triathlon (Anderson et al. 2018; Henst et al. 2015; Kunorozva et al. 2012). Indeed, Lastella et al. (2016) and Kunorozva et al. (2012) observed that only a few evening type athletes are involved in endurance sports with regular morning sessions. Interestingly, it has been suggested that habitual training time could influence physical performance regardless of chronotype (Rae et al. 2015; Vitale and Weydahl 2017), however, available findings are mixed. Some studies have observed significant diurnal differences in peak performance between chronotypes even though athletes regularly trained in both morning and evening sessions (Anderson et al. 2018; Martin et al. 2007). In contrast, others have observed that chronotype effects are diminished in athletes who undergo regular morning or evening sessions (Chtourou et al. 2012; Chtourou and Souissi 2012; Rae et al. 2015). As it is currently unclear if regular training during the minima of the diurnal cycle influences training adaptation, coaches may want to consider diverse or flexible training schedules to reduce their physiological stress and potentially increase performance capability during training.

## 5 Limitations

It is acknowledged that the sample size within the present study is slightly below the calculated optimum. However, within the present study we deliberately limited the population to the two extreme chronotypes, maximising potential differences. Considering the theoretical influence of chronotype on performance is plausible (Lastella et al. 2016; Vitale and Weydahl 2017) and that literature on endurance performance is scarce we think the current results can still be relevant for sport science research and practise. Due to the sample size the current results should be interpreted with caution and future research should evaluate if the results are reproducible with larger sample sizes and across a variety of sports. Interestingly, it was especially difficult to find an adequate number of evening‐type athletes which might be due to the chronotype distribution in the population. Several studies reported a bell‐shaped distribution with the majority of people being classified as intermediate types and fewer individuals defined as morning‐ or evening‐types (Adan and Natale 2002; Kabrita et al. 2014). Indeed, we originally approached 70 competitive cyclists of which only seven could be classified as clear evening types. This slightly uneven distribution is in accordance with previous research observing more morning‐type athletes in sports like cycling, running and triathlon (Henst et al. 2015; Lastella et al. 2016; Rae et al. 2015). If athletes pursue and excel in sports that match their chronotype with regular early morning sessions leading to a drop‐out of evening‐type athletes or if chronotype may be modified by behaviour is yet unknown. Additionally, with increasing age, daily preferences and activities shift towards morningness, including earlier bed‐ and waking times (Brown et al. 2008; Roenneberg et al. 2004). Considering the mean age of participants in this study (37.9 years) this might have further affected the difficult recruitment of evening‐type athletes. It should be noted that rising at 06:30 h may favour morning‐type individuals. However, only participants with habitual wake‐up times between 06:00 and 07:30 h were included, and all had regular morning commitments (e.g., school, work, university), making this schedule typical. Whilst evening‐type athletes may prefer later rising times, they often must adapt to externally determined training and competition schedules. As wake‐up times were kept consistent across the study regardless of testing time, it appears unlikely that this factor systematically influenced the results.

Given the connection between chronotype, wellbeing, and mood states, the use of questionnaires such as wellness scales or the POMS would have been useful to further explore links between time‐of‐day predispositions and performance. However, to reduce participant burden, only short VAS ratings of motivation, sleepiness, and readiness were collected.

Body temperature is generally considered to be one of the primary endogenous indicators of the diurnal rhythm of individuals (Vitale and Weydahl 2017). Based on that, core body temperature was measured via electronic pills; however, consistent data was only available from five participants, thus the data could not be included in the final analysis.

## 6 Conclusions

In conclusion, the results of the present study indicate that an individual's chronotype might have an influence on the timing of their peak cycling performance throughout the day. Indeed, the results show that evening type athletes performed better later in the day. The different observed responses of morning and evening type athletes appear in part to be associated with differences in perceived evening mental readiness and morning sleepiness. Future research should investigate if the observed negative effect of this study is reproducible and consistent within and between athletes and sports. Furthermore, if and how the individual chronotype might influence the training response and if someone's chronotype can be changed (e. g. through habitual training time) remains unknown.

## Author Contributions

**Sabrina Forster:** conceptualization, writing – original draft, investigation, methodology, visualization, formal analysis, data curation, supervision, project administration. **Sascha Schwindling:** conceptualization, investigation, methodology, project administration. **Chris Abbiss:** writing – review and editing, resources. **Fabienne Döringer:** data curation, writing – review and editing, investigation. **Andreas Klütsch:** investigation, writing – review and editing, data curation. **Anne Hecksteden:** writing – review and editing, methodology, data curation, supervision. **Tim Meyer:** writing – review and editing, supervision, resources.

## Funding

The authors have nothing to report.

## Ethics Statement

Due to ethical restrictions, some data may not be publicly available. However, anonymized datasets and relevant supporting materials can be shared upon request and subject to institutional approval or data use agreements. All data generated or analysed during this study are included in this published article. No disclosure of a conflict of interest to state for any of the authors. No funding was received for this study. The study was conducted in accordance with the Declaration of Helsinki and was approved by the local Human Research Ethics Committee (Ärztekammer des Saarlandes, Saarbrücken, Germany). Before testing, all participants gave written informed consent and provided details on their training and racing history.

## Conflicts of Interest

The authors declare no conflicts of interest.

## Supporting information


**Table S1:** Checklist of considerations in chronobiological studies on human and sporting performance for participant (1–3), methodological and equipment (4–8) and environmental (9) considerations and general comments and insights into decisions for our study.

## Data Availability

The data that support the findings of this study are available from the corresponding author upon reasonable request.
