# Supplementary material for: Influence of Chronotype on Cycling Performance in Simulated 20‐km Time Trials—A Pilot Study
Source: J Sleep Res. 2025 Dec 16;35(3):e70268. doi: 10.1111/jsr.70268 (PMC13193443; doi:10.1111/jsr.70268)
Supplement: Supplementary file 1 — Table S1: Checklist of considerations in chronobiological studies on human and sporting performance for participant (1–3), methodological and equipment (4–8) and environmental (9) considerations and general comments and insights into decisions for our study. [file JSR-35-e70268-s001.docx]

**SUPPLEMENTARY TABLE 1** Checklist of considerations in chronobiological studies on human and sporting performance for participant (1 – 3), methodlogical and equipment (4 – 8) and environmental (9) considerations and general coments and insights into decisions for our study

| Consideration | General commenst and insights into the decision for our study |
| --- | --- |
| (1) *Sample size estimation and rational* | A-**priori test** completed with power levels of 80%, p = 0.05 and meaningful effect size given – either from Cohen d tables or past literature with the reference for the research. This should be reported in the methods, in the statistical analysis section. It has been suggested that authors should report the full range of information required to enable the sample size estimation and rationale to be examined. This includes software used, the exact inputs to calculations, a rationale for those inputs, stopping rules and the statistical tests used to test a hypothesis or estimate a population parameter (Abt et al., 2020) – *we conducted this and based the effect size for the sample size calculation on the mean difference between (md) the 1^st^ and 4^th^ place during the German Road Cycling Championship between 2019-2021 (md: 66.5 s) and the variability of laboratory based cycling to consider practical relevance for trained athletes* |
| (2) *Population* | **Biological sex**: The combination of male and female data adds complexity to the interpretation of results and for the protocol we used add months to the data collection timeframe. **Chronotype** and **sleep habits** should be reported and interpreted (Walsh et al., 2021) – *for our study where there is disagreement in a diurnal variation, we sought to limit variation in our population and only used male, intermediate participants. Sleep habits were monitored for three consecutive nights prior* to data collection. Categorising **fitness** (such as by McKay et al.(2022)) and markers of fitness should be done to help define the populations such as VO_2max/peak_ or Wingate tests. This information helps with further analysis depending on the distance of the time-trial and contribution of anaerobic or aerobic pathways - *we used the methods of (McKay et al.(2022). As markers for fitness we uses maximal power output (P_max_).* |

**SUPPLEMENTARY TABLE 1** (Continued)

| Consideration | General commenst and insights into the decision for our study |
| --- | --- |
| (3) *Inclusion/exclusion criteria to reduce confounding variables in studies* | **Inclusion**: No diagnosed **sleep disorders**; have not completed **shift work** or **travelled** outside the local time-zone in the past month; **injury-free**; habitually **train** at any time of day; habitual **total sleep time** appropriate to age and **retiring and protocol waking times** within that of the participants **normal** hygiene; **not** receiving **pharmacological treatment** (including **NSAIDs**); a habitual **caffeine** “low” consumption, hence <**150 mg per day** assessed by the caffeine consumption questionnaire (Drust et al., 2005; Yousefzadehfard et al., 2022) – *we included these points in the method section*. |
| (4) *Allocation of IDs to session* | Either **randomly allocated** to groups (randomised) and then the order of sessions for groups **counterbalanced** in order of administration to minimise any potential learning effects (Monk and Leng, 1982). This is sometimes referred to as cross over design or a cyclic Latin design (Drust et al., 2005). Or, **allocated into two groups (named 1 and 2)** equally based on physical ability for first and second session allocation. Practically entails stacking the performance measure for the last familiarisation session from fastest to slowest in Microsoft Excel, then pasting the words 1 or 2 respectively so the first cell (fastest participant) was 1 and next was 2 all the way down the column - *we included these points in the method section*. |
| (5) *Timing of session and time between sessions* | Being as close to the body temperature minimum and maximum as possible to maximise the chance of finding a rhythm (04:00 and 16:00 h respectively), but not so early as to cause sleep deprivation in the morning or be beyond the opening hours of the research facility; so 06:00–07:30 h in the morning and 17:00–19:00 h in the evening timings are normally chosen for diurnal studies - *we followed this logic with the exception of the 22:00 h trial. As elite athletes regularlay have to perform after 19:00 h, we think evaluating potential effects on performance later at night is important for translating the results into sports practice.* |

**SUPPLEMENTARY TABLE 1** (Continued)

| Consideration | General commenst and insights into the decision for our study |
| --- | --- |
| (6) *Familiarisation of participantsto the performance test* | The rational for the **number of familiarisations** should be given as well as random and systematic bias of the last and penultimate given in the method section. This then quantifies the level of learning in the population of the research under investigation. Furthermore, familiarisation is an important experimental methodological consideration for sport and exercise scientists, since it may influence both the number of experimental trials needed in a study, and the sensitivity of the exercise performance criteria. It is therefore potentially very useful to know the test-retest reliability of performance measurements that are used in this manner - *we included these points in the method section*. |
| (7) *Diet (timing/content) and timing of food consumption relative to the session* | The macro and micro-**diet** weekly habits given with normal caffeine consumption. Participants recorded the type, amount, and timing of the food they ate for the period of 24 h before the day of the first session and were asked to replicate this diet for the days before the second experimental condition. Morning participants tend to come in fasted and evening not having eaten 4-h prior (Drust et al., 2005). Where is the difference in food intake is 3–4 h between overnight fast and evening fasting - we adopted a pragmatic approach where there was no consensus and having a breakfast or not in the morning had no effect on a range of performance measures (Bougard et al., 2009) – *we followed this logic.* |

**SUPPLEMENTARY TABLE 1** (Continued)

| Consideration | General commenst and insights into the decision for our study |
| --- | --- |
| (8) *Method of temperature measurement for core body temperature and ergometers chosen for the time-trial* | The hierarchy for the measurement site of “core” temperature being Oesophageal, gut, rectal, intra-aural and oral sites for rest, with only oesophageal, gut, rectal sites used at during exercise (Waterhouse et al., 2005). The factory error associated with the ergometer given as well as laboratory internal calibration checks. Consideration such as can the system measure distance or do they make approximate assumptions should be given - *we followed this logic and used, Cyclus 2 ergometer has a ±2% error in measuring distance and power. Core body temperature was measured via electronic pills, however consistent data was only available from five participants* |
| (9) *Time of year and season given. Laboratory conditions* | Inclusion of season(s) and range of time of dawn and dust hence total daylight hours should be given. Also, information on exposure to light in the morning session, both in the laboratory and before should be recorded. The range of ambient lighting, dry temperature, relative humidity, and barometric pressure should be given in method section - *we followed this logic*. |
